# Feeding-induced hepatokines and crosstalk with multi-organ: A novel therapeutic target for Type 2 diabetes

**DOI:** 10.3389/fendo.2023.1094458

**Published:** 2023-03-03

**Authors:** Rong-Bin Chen, Qi-Yu Wang, Yuan-Yuan Wang, Ya-Di Wang, Jiang-Hua Liu, Zhe-Zhen Liao, Xin-Hua Xiao

**Affiliations:** ^1^ Department of Metabolism and Endocrinology, The First Affiliated Hospital, Hengyang Medical School, University of South China, Hengyang, Hunan, China; ^2^ Department of Clinical Laboratory Medicine, Institution of Microbiology and Infectious Diseases, The First Affiliated Hospital, Hengyang Medical School, University of South China, Hengyang, Hunan, China

**Keywords:** type 2 diabetes, insulin resistance, glucolipid metabolism, feeding-induced hepatokines, multi-organ

## Abstract

Hyperglycemia, which can be caused by either an insulin deficit and/or insulin resistance, is the main symptom of Type 2 diabetes, a significant endocrine metabolic illness. Conventional medications, including insulin and oral antidiabetic medicines, can alleviate the signs of diabetes but cannot restore insulin release in a physiologically normal amount. The liver detects and reacts to shifts in the nutritional condition that occur under a wide variety of metabolic situations, making it an essential organ for maintaining energy homeostasis. It also performs a crucial function in glucolipid metabolism through the secretion of hepatokines. Emerging research shows that feeding induces hepatokines release, which regulates glucose and lipid metabolism. Notably, these feeding-induced hepatokines act on multiple organs to regulate glucolipotoxicity and thus influence the development of T2DM. In this review, we focus on describing how feeding-induced cross-talk between hepatokines, including Adropin, Manf, Leap2 and Pcsk9, and metabolic organs (e.g.brain, heart, pancreas, and adipose tissue) affects metabolic disorders, thus revealing a novel approach for both controlling and managing of Type 2 diabetes as a promising medication.

## 1 Introduction

Diabetes is a major illness worldwide that is reaching an epidemic stage (1). Approximately 500 million individuals across the globe are living with diabetes, and it is anticipated that this figure will rise by 25% by the year 2030, and by 51% (roughly 700 million people) by the year 2045 (2). As a result, ensuring that the disease is avoided and effectively treated is an ultimate importance to public health. Most cases of type 2 diabetes mellitus (T2DM) are caused by modifiable risk factors such as diet (3). Epidemiological surveys have found that the incidence of eating disorders in individuals with type 2 diabetes ranges from 1.2% to 14%, mainly manifesting as over-eating (4). Prior research has declared that nutritional behavior is mainly related to neural regulation in the brain (5). However, recent studies have shown that the liver maintains systemic metabolic homeostasis by transcriptionally controlling the expression of open organ factors in response to external signals such as feeding behavior (6).

In T2DM, when insulin secretion is insufficient, it first causes impaired glucose metabolism. Glycogen synthesis is reduced, and catabolism is increased, tissues’ ability to take up and use glucose is reduced, which triggers fasting and postprandial hyperglycemia (7). The malfunction of glucose oxidation and the enhancement of lipolysis metabolism result in an elevation in free fatty acids in the blood, which enter the liver and promote synthesis and release of triglycerides and very-low-density lipoproteins (VLDL), resulting in disorders of lipid metabolism (8). Therefore, it is essential to regulate the metabolism of glucose and lipid in diabetic patients and to understand the treatment mechanism of diabetes, thus improving T2DM patients’ life quality.

The liver is a vital organ in the modulation of energy homeostasis because it detects and reacts to shifts in the nutritional condition that occur in response to a wide range of metabolic circumstances (9). The majority of the attribution for the liver’s function in the modulation of systemic glucolipid metabolism goes to the release of hepatokines that maintain metabolic homeostasis through autocrine, paracrine and endocrine pathways that regulate the connections between the liver and other organs (10). Of note, feeding can induce the release of hepatokines, which can act on other organs to influence the development of diabetes. Mechanistically, based on the available literature, these feeding-induced hepatokines act through one or more of the following metabolic organs (1) improving pancreatic β-cell cholesterol accumulation, reducing endoplasmic reticulum stress, (2) reducing white adipose tissue inflammation and inhibiting lipid accumulation, (3) inhibiting the brain feeding center and regulating energy homeostasis (**Supplemental Table 1**).

**Figure 1:**
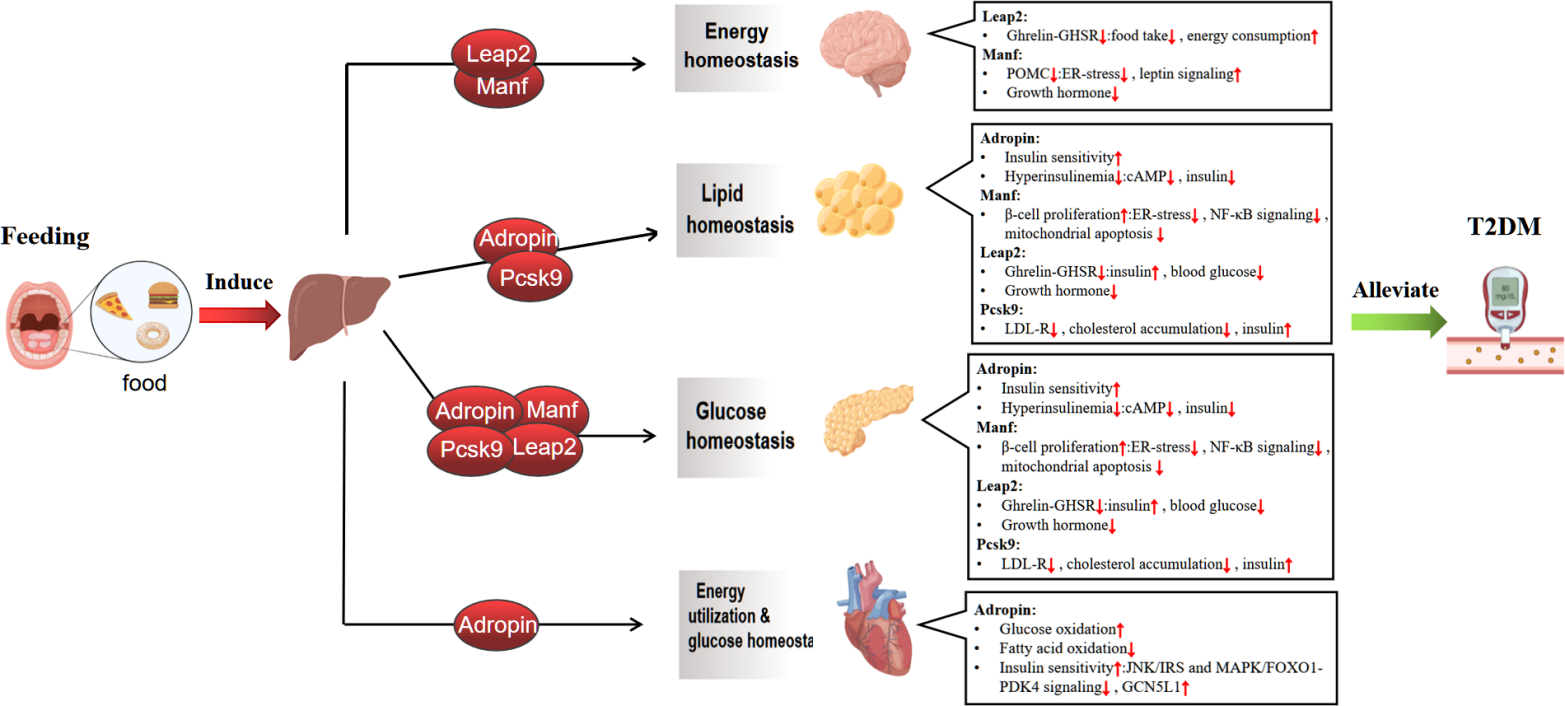
Mechanisms of feeding-induced hepatokines amelioration of Type 2 diabetes in target tissues. The liver plays a central role in regulating systemic metabolic homeostasis by sensing nutrient availability and altering metabolite and energy production for use by various organ systems. The act of feeding induces the liver to release hepatokines, which regulate glucolipid metabolism and maintain energy balance by affecting multiple metabolic organs, including the brain, adipose tissue, pancreas and heart, thereby improving type 2 diabete.↑ increase, ↓ decrease. Graphics from http://Biorender.com.

In this review, We focus on the feeding-induced hepatokines, including Adropin, Manf, Leap2 and Pcsk9, Which participate in the occurrence and development of diabetes. We also highlight the potential mechanisms by which these hepatokines mediate crosstalk between the liver and other organs (brain, heart, adipose tissue, and pancreas) and the possibility of using them as new treatments for T2DM (**Figure 1**).

## 2 Adropin

Adropin was originally thought to be a liver-derived peptide implicated in both the homeostasis of energy and the metabolism of glucolipids. The energy homeostasis-related (ENHO) gene encodes a 43-amino acid polypeptide (residues 34-76) that is used to produce this factor (11). There is a possibility that adropin functions as a secretory product of the hepatic biological clock, coordinating metabolic and circadian rhythms and responding to a large number of nutrients and energy balances in the diet. According to preliminary studies, sufficient energy is required for adropin expression in the liver (12). DIO decreased adropin levels in the serum, which were elevated in the nutritional condition (12, 13). As per the current work, the highest expression of Adropin in mice was associated with transcriptional stimulation by RORα/γ, while the minimum expression was associated with Re-verb transcription. Small molecules influencing Rev-erb blocking ability and transcriptional stimulation by RORα/γ quickly modified the expression of ENHO in human HepG2 cells (14). Impressively, it was observed that a high-fat diet that enhances hepatic Adropin expression also elevates liver ROR expression (15). Participation in these nuclear receptors also appears to offer a feasible mechanism for nutritional sensing in the modulation of Adropin expression. Although the link between plasma Adropin levels, nutrition, and mealtime is lesser understood in people, plasma Adropin levels in nonhuman primates (constant monkeys) have also peaked at feeding times in available studies (15). In addition, levels of serum Adropin were decreased in patients with T2DM, and elevated circulating Adropin values are related to a decreased hazard of diabetes-related complications (16–18). Here, we summarize the crosstalk of feeding-induced hepatokines on multiple extra-liver organs (i.e., adipose tissue, pancreas, brain, and heart). We hypothesize that it is participated in the mechanisms of reduced obesity, insulin resistance, and glucolipid metabolism, thereby alleviating T2DM glycolipotoxicity.

### 2.1 Impact of Adropin on energy metabolism

The heart of diabetic patients relies more on the oxidation of fatty acids for producing energy and exhibits impaired glucose uptake and insulin signaling. These changes in cardiac metabolic activity of energy play a part in heart illness intensity (19, 20). Adropin induces significant alterations in cardiac energy metabolic activity *via* boosting glucose metabolism and inhibiting fatty acid oxidation while inhibiting the JNK/IRS-1 S307 phosphorylation axis improves insulin sensitivity, hence boosting metabolic statuses and cardiac efficiency (21). Adropin is hypothesized to have an insulin-sensitizing action to reduce the downregulation of pyruvate dehydrogenase (PDH) negative regulator PDK4 expression levels through MAPKs and FOXO1 signaling mechanisms (21, 22) or modulates the expression of the mitochondrial acetyltransferase GCN5L1, which alters the acetylation condition and the energy activities of the metabolizing enzymes to promote glucose oxidation (23). It also inhibits fatty acid uptake in muscle membranes and mitochondria at the transcriptional level by reducing the protein levels of fatty acid transporter CD36 and carnitine palmitoyltransferase 1 (CPTI) (24, 25). These findings show that adropin plays a significant part in modulating the preference for cardiac energy substrates. In clinical practice, low levels of the serum protein Adropin are connected with many cardiovascular diseases like endothelial malfunction (26–28), heart failure (29, 30), acute myocardial infarction (31), coronary atherosclerosis (32–34), and type X heart syndrome (35). Low levels of Adropin also serve as a separate threat variable and indicator for most illnesses. As a result, there is a good chance of a connection between the levels of Adropin and diabetes-related cardiovascular illness and energy metabolism. This connection needs to be fully investigated.

### 2.2 Effect of Adropin on lipid metabolism

Previous studies demonstrated that adropin could be participated in the control of adipose tissue function. Adrpin overexpression delayed weight gain in mice fed high-fat meals compared with wild-type animals (11). *In vitro*, through ERK1/2 and AKT-dependent signaling, adropin stimulates the growth of 3T3-L1 cells and mice preadipocytes. Additionally, adropin reduces the lipid deposition and expression of lipogenic genes in these cells. (Pparγ, Fabp4, C/ebpα), bringing about a final reduction in their differentiating process to mature adipocytes (36). Similarly, adropin stimulated brown adipose tissue (BAT) preadipocyte proliferation in Wistar rats through an AKT-dependent pathway, but inhibited preadipocyte maturation by downregulating lipogenic genes (C/ebpα, C/ebpβ, Pgc1α, Pparγ, and Prdm16). Additionally, this study found that adropin decreased lipid accumulation in BAT and increased glycerol and free fatty acid release. It also promoted hormone-sensitive lipase (HSL) activity (37). Of note, the hormone network is complex, and in addition to acting directly on the liver, hormones can interact with other hormones to regulate metabolic homeostasis. Recent studies have shown that adropin slightly promotes lipolysis in rat adipocytes and 3T3-L1 cells but does not affect glucose uptake. In addition, adropin may exert an ameliorative insulin resistance and anti-inflammatory effect by upregulating the expression of adiponectin and inhibiting the expression of resistin and visfatin (38, 39). Overall, Adropin inhibits adipogenesis as well as intracellular lipid accumulation, suggesting that it may improve diabetes by regulating lipid metabolism in adipose tissue as well as modulating the release of other adipokines. Although to further understand the function of Adropin in regulating adipose metabolic mechanism and the development of adipose tissues *in vivo*, additional research is required.

### 2.3 Effect of Adropin on glycolipid homeostasis

Adropin depletion is linked to higher intensity of glucose homeostasis imbalance as well as abnormalities of lipid metabolic activities when observed *in vivo*. A functional investigation of Adropin knockout (AdrKO) was carried out in C57BL/6J mice by Chen et al. The findings demonstrated that WT mice had normal blood glucose levels significantly lower than those of AdrKO mice when given a conventional diet for one year (P < 0.0001). It is interesting to note that after 30 weeks, almost all AdrKO mice developed T2DM when subjected to a high-fat initiation and impaired glycosphingolipid biosynthesis. In addition, a significant number of adipocytes infiltrated the pancreas, a hallmark of a fatty pancreas (FP) (40). Furthermore, the serum levels of Adropin were shown to be significantly reduced in individuals with FP and T2DM compared with healthy individuals, and the levels of relative modulatory T cells (Treg) were also found to be significantly lower and positively connected with Adropin levels (r=0.7220, P=0.0001) (40). Furthermore, the serum levels of Adropin were shown to be significantly reduced in individuals with FP and T2DM compared with healthy individuals, and the levels of relative modulatory T cells (Treg) were also found to be significantly lower and positively connected with Adropin levels (r=0.7220, P=0.0001) (40). Treg functions as a negative modulator of the inflammatory condition of adipocytes and were discovered to minimize IR, thereby controlling insulin sensitivity (41). In a model animal with IR caused by a high-fat diet, Adropin can reduce insulin mRNA expression and secretion by affecting the synthesis of cyclic adenosine monophosphate (cAMP) in pancreatic cells without affecting β-cell viability or proliferation (42). Overall, these findings point to the possibility that Adropin enhances insulin sensitivity and reduces IR by altering T_reg_ number or function and modulating insulin secretion.

## 3 Manf

Although midbrain astrocyte-derived neurotrophic factor (Manf) was basically classified as a neurotrophic indicator, the protein does not structurally or functionally resemble a true neurotrophic factor. Neurotrophic factors act by the interaction with similar receptors found in the plasma membrane, although no cell surface receptors for Manf were identified (43). Recently Wu et al. demonstrated that RNA sequencing investigation of the livers of mice that were fasted and then fed revealed that Manf is a feeding-induced hepatokine. Manf, which is generated from hepatocytes, raises the body’s rate of energy consumption, which combats diet-induced obesity. It would indicate that Manf is directly responsible for the browning of white adipose tissue (WAT) in the groin^13^. Under typical circumstances, the protein Manf can be found in the lumen of the endoplasmic reticulum (44). Endoplasmic reticulum stress (ERS) can increase its expression in different cells and tissues (44–46). The aggregation of unfolded or misfolded proteins in the endoplasmic reticulum causes ER-stress (47). It does this by activating a cellular defensive reaction known as the unfolded protein response (UPR). This response is a signaling cascade that restore endoplasmic reticulum stability (48). It is interesting to note that Apostolou et al. confirmed that Manf is a UPR gene that is able to reduce the amount of apoptosis caused by endoplasmic reticulum stress (46). Moreover, the serum levels of Manf were shown to be lower in patients who had T2DM and had a correlation with the metabolism of lipids and glucose (49). Here, we hypothesize that Manf may mitigate the progression of T2DM by modulating lipid metabolism, inflammation, apoptosis, and proliferation in the liver, adipose, and pancreatic tissues.

### 3.1 Effect of Manf on glucose metabolism

Increasing evidence suggests that if endoplasmic reticulum stress is not resolved, the UPR transitions from an adaptive (A-UPR) response to a prolonged unresolved UPR, which ultimately results in enhanced inflammatory signaling and autophagy, and apoptosis (50, 51). This is the main cause of β-cell malfunction and death in T2D (52). In T2DM, β-cells are subjected to local environmental parameters, including glycolipotoxicity and inflammatory cytokines, which results in impaired insulin synthesis and increased free fatty acid production, as well as unresolved cell endoplasmic reticulum stress and β-cell death (53, 54). The stimulation of the UPR, which is connected to the buildup of lipid metabolites, is also connected, in a pathological manner, with IR in specific tissues (54). Of interest, most of the literature suggests that the IRE1/XBP1 and ATF6 pathways are involved in the key function of Manf in attenuating the negative modulation of UPR by endoplasmic reticulum stress (45, 55–57). The latest research demonstrated that hepatocyte-derived MANF plays a crucial role in increasing insulin sensitivity and that the systemic injection of MANF protein greatly enhanced insulin sensitivity in mice exhibiting obesity (58). Besides insulin sensitivity, Manf promotes insulin secretion by maintaining the number of pancreaticβ-cell. Ablation of MANF in mouse embryos, both overall and in the pancreas, leads to early onset and severe diabetes mellitus. This is because in Manf -/- mice, phosphorylation of eIF2α inhibits the translation of cyclin D1 and the cell cycle is subsequently arrested in the G1 and G2/M phases, ultimately leading to reduced β-cell proliferation and increased apoptosis (59, 60). MANF overexpression promotes the growth of primary β-cells in humans and mice having diabetes, as well as protection of people and mouse β-cells from the death induced by endoplasmic reticulum stress in β-cells to some extent (61–63). The protective and proliferative effects of MANF on β-cells were correlated with the suppression of NF-κB signaling pathway and amelioration of endoplasmic reticulum stress as well as blocking BH3-only proteins BIM-dependent triggering of mitochondrial apoptotic pathway (64). Chen et al. found that MANF can interact with the DNA binding domain of p65 through its C-terminal SAP-like structural domain and is a key target gene for inhibiting NF-κB signaling pathway (65). Later, Yagi et al. reported that Neuroplastin (NPTN) is a plasma membrane receptor for MANF. The binding of MANF to NPTN attenuates the inflammatory reaction and cell death by inhibiting the NF-κB signaling pathway (66). Another study showed that MANF attenuates endoplasmic reticulum stress by suppressing the IRE1-caspase 12-caspase 3 cell death pathway and has a protective effect against pancreatic alveolar cell injury (67). These results are potential mechanisms for the protective and proliferative influences of MANF on β-cells, which perform important implications for the modulation of insulin production and improvement of glucose metabolism. Notably, Montaser et al. identified the MANF pure-hybrid loss-of-function mutation as a novel gene causing diabetes and neurodevelopmental disorders in children (68). In conclusion, these data further support that MANF performs a crucial part that helps pancreatic β-cell s to survive and proliferate and hence could provide a possible therapy for T2DM patients.

### 3.2 Effect of Manf on lipid metabolism

Manf differs from any known nerve growth factor in that its N-terminal structural domain is saposin-like lipid conjugation domain (69). SAPLIPs (saposin-like proteins) are a family of lipid-interacting proteins that vary in size and activity and have a wide variety of cellular functions (70). Bai et al. proposed that Manf binds to lipid sulfolipids, which are called 3-O-sulfogalactosyl ceramides, a lipid that exists in the outer leaflet of serum and cell membranes (71).Thus, Manf can bind lipids. Notably, Sousa-Victor et al. identified Manf as a stress response protein that is released and has immunomodulatory effects, as well as being part of an evolutionarily conserved system and a controller of the hepatic metabolic homeostasis in particular (72). Manf heterozygous mice showed an inflammatory phenotype in multiple tissues, and hepatocellular steatosis and fibrosis, besides developing hepatic bone disease at a faster rate than control mice (72). Overexpression of Manf was able to rescue HepG2 cells from the steatosis that was caused by free fatty acids (FFAs). This was accomplished by inhibiting the synthesis and uptake of fatty acids, as well as suppressing the synthesis of cholesterol. Thus, Manf inhibited lipid deposition in HepG2 human hepatocytes (73). Furthermore, an increase in the levels of the autophagy markers LC3-II and Atg5 was responsible for the attenuation of hepatic steatosis in mice that had Manf overexpressed, which is liver-specific. In addition to this, Manf was responsible for a rise in the phosphorylation of Stat3 as well as its nuclear localization (74). Therefore, Manf influences the metabolism of hepatic lipids by controlling autophagy. The overexpression of genes linked to lipid metabolism, in particular G0/G1 Switch gene 2 (G0S2), appears to be associated with the negative effect of diminished Manf expression in the liver. G0S2 is an important modulator of lipid metabolism and act as a suppressor of lipolysis. It was demonstrated that knocking down Manf causes higher levels of G0S2, which in turn causes hepatic steatosis as well as a pro-inflammatory state in macrophages (72, 75). Knockdown of Manf resulted in an increase in the production of TNF-α, IL-1α, and IL-6 (76). In addition, the increased expression of Manf, which is liver-specific prevented obesity in mice caused by a high-fat diet and accelerated browning of white adipocytes through activating the P38 MAPK pathway. Elevating the expression of key lipolytic proteins (phosphorylated hormone-sensitive lipase (HSL) and adipose triglyceride lipase (ATGL)) is how Manf impedes the expression of M1-type macrophage polarization indicators in mouse eWAT. This assists in decreasing adipose inflammation and improving insulin sensitivity and lipid deposition in high-fat-fed mice (58). In conclusion, these data further support that MANF can improve lipid metabolism in T2DM by a down-modulating inflammatory reaction and lipid deposition in the liver and adipose tissues.

### 3.3 Effect of Manf on energy metabolism

MANF influences food intake and energy balance by regulating hypothalamic insulin signaling, suggesting that MANF-mediated neuronal activity plays an important role in maintaining energy homeostasis. Furthermore, MANF is enriched in different nuclei of the mouse hypothalamus and critically regulates energy intake, but energy expenditure seems to be unaffected (77). It has been shown that high levels of MANF expression in the rat hypothalamus persist into adulthood (78), raising the possibility that MANF plays an important role in the mature hypothalamus. It is known that ER stress in the hypothalamus leads to leptin resistance and hyperphagia (79), whereas MANF overexpression in Hypothalamic pro-opimelanocortin (POMC) attenuates ER stress and leads to increased thermogenesis in the BAT by improving leptin signaling in the hypothalamus and regulating sympathetic innervation and activity in it (80). These results suggest that MANF overexpression in the hypothalamic nucleus leads to severe hyperphagia and obesity. However, MANF can properly regulate energy homeostasis through POMC neurons. Furthermore, MANF appears to have multifaceted and cell type-specific functions, as recombinant human MANF was recently found to promote corneal epithelial wound healing and nerve regeneration in diabetic patients by attenuating hyperglycemia-induced endoplasmic reticulum stress through the Akt signaling pathway (81). MANF may be a useful therapeutic modality in the treatment of diabetic keratopathy (DK). A recent study showed that strong expression of MANF was also observed in the mouse pituitary, thyroid, and adrenal glands, all tissues involved in the neuroendocrine axis, and important for the regulation of feeding, stress, growth, and development. Interestingly, compared to wild-type mice, MANF-deficient mice have smaller anterior pituitary lobes and reduced numbers of cells producing growth hormone (GH) (82). GH has also been described as a diabetogenic agent with the ability to reduce insulin sensitivity (83). In the brain, GH activates the expression of AgRP neurons, increasing food intake while decreasing energy expenditure (84, 85). These results suggest that MANF plays an essential role in highly hormone-secreting cells within the hypothalamic-pituitary-thyroid/adrenal/gonadal axis and that proper regulation of MANF expression in the brain and other endocrine organs is vital to meet metabolic demands.

## 4 Leap2

Liver expresses antimicrobial peptide 2 (Leap2), a bicyclic cationic polypeptide (86). As a feeding-induced hepatokine, it is highly expressed in the liver, and its release is inhibited by stopping feeding and returns to baseline levels after subsequent feeding (87). Ghrelin is a hormone that stimulates hunger and is released by the stomach. Ghrelin’s function is modulated by binding to the growth hormone secretagogue receptor (GHSR) (88). The ghrelin-GHSR system is implicated in a wide range of biological activities, including the enhancement of growth hormone (GH) production, enhanced hunger and food consumption, control of glucose homeostasis, and cardiovascular function (89–92). According to the most recent findings, Leap2 and Ghrelin are paired with other factors in a competing way with GHSR (93). Thus, Leap2 is a competitive antagonist of GHSR, rather than a non-competitive antagonist as previously reported (94). Leap2 prevents the principal activities of Ghrelin *in vivo*, such as food consumption, GH release, and the control of survivable blood glucose levels throughout periods of calorie reduction or fasting. On the other hand, inhibiting Leap2 has the effect of amplifying the actions of ghrelin (94, 95). As a result, it appears that Leap2 may perform a significant part in metabolic illnesses by acting as a modulator of the ghrelin-GHSR system. In addition, patients who have T2DM had lower serum levels of ghrelin and higher serum levels of Leap2. It’s possible that the interaction between Ghrelin and Leap2 performs a significant part in the progression of T2DM. There is some speculation that the ghrelin-Leap2 axis could be a viable therapeutic target for T2D (96). In conclusion, we came to the view that Leap2 has the possibility to be an applicable treatment for the control of T2DM. This is because the ghrelin-GHSR system, which modulates energy metabolism in the brain, also modulates glucose metabolism in pancreatic tissue.

### 4.1 Effect of Leap2 on energy metabolism

Furthermore, the N-terminal region alone delivers binding and activation to the Leap2 receptor. Leap2 and its N-terminal part were discovered to act as an inverse agonist of GHSR and also a competing antagonist of Ghrelin-induced phosphatidylinositol production and calcium mobility. Both inverse agonists and antagonists act on the agonist but interact with the receptor in different ways. The inverse agonist binds to the same receptor as the agonist but brings about the opposite response to the agonist, while the antagonist binds to the receptor and disrupts the interaction and function of the agonist and counter-agonist at the receptor (97). LEAP2 is both an inverse agonist of GHSR, which downregulates the constitutive activity of GHSR, and a competitive antagonist, which impairs gastrin-induced activation of GHSR. Thus, Leap2 exerts its inhibitory effect on the ghrelin-GHSR system through its N-terminal region (95). On the one hand, Ghrelin signals are transmitted *via* the vagus nerve to the hypothalamus, which is the modulatory center of nutritional behavior (90). Islam et al. declared that intracerebroventricular (i.c.v.) injection of Leap2 into mice was shown to suppress central Ghrelin function, such as hypothalamus nucleus Fos expression, promoted feeding, elevated blood glucose, and lowered body temperature. However, intraperitoneal (i.p.) leap2 administration showed no reduction in neuropeptide Y (NPY)-induced food consumption or des-acyl ghrelin-induced inhibition in body temperature, demonstrating that Leap2’s suppressing activity is specific to the GHSR (98). In contrast, GHSR was strongly expressed in the arcuate nucleus of the hypothalamus (ARC), the dorsal medial nucleus of the hypothalamus (DMH), the ventral medial nucleus of the hypothalamus (VMH), and the lateral hypothalamic nucleus (LH) (99). GHSR governs essential physiological activities such as hunger, neuroendocrine axis, autonomic nervous system activities, and sophisticated mental processes like reward-related attitudes (100). Therefore, the primary function of GHSR is the modulation of neuronal activities (101). The voltage-gated calcium channel 2.2 (Cav2.2) is a prominent GHSR target in neurons. Heterologous expression systems and membrane clamp recordings suggest that the N-terminal region of Leap2 binds GHSR, thereby impairing the ghrelin-dependent (GQ protein signaling) and ghrelin-independent modes of GHSR action (Gi/o protein activation) on the suppression of Cav2.2 currents (102). In addition, the N-terminal region of Leap2 also affects the inhibitory modulation of Cav2.2 currents by the heterodimer of GHSR-the dopamine 2 receptor (D2R)-and its coupling to G proteins (103). Cornejo et al. declared that intracerebroventricular (i.c.v.) injection of C57BL/6J mice with an N-terminal Leap2 fragment diminished overeating in mice on a high-fat diet (104). Taken together, The N-terminus of Leap2 inhibits the ghrelin-GHSR pathway in the central nervous system. Contrarily, the elimination of the LEAP2 gene raised weight gain, food consumption, lean body mass, and liver adipose tissues in HFD-fed female rats. This is a result of less energy consumption, decreased physical exercise, and increased food consumption (105). Furthermore, the Ghrelin-AMPK-SREBP1 pathway may modulate the expression level of Leap2 in the liver. Through the hepatic-gastric-brain axis (98), Leap2 may impact eating and energy balance. Latest research demonstrated that Leap2 also mediates the outcomes of food consumption and energy metabolism through the endogenous cannabinoid system (eCBome)-gut microbiome (mBIome) axis (106). In summary, LEAP2 can improve diabetes by inhibiting brain intake-related energy metabolism.

### 4.2 Effect of Leap2 on glucose metabolism

One of the main features of Ghrelin and LEAP-2 is that they have opposite effects on GH secretion (107). A recent study using two animal models of GH deficiency found a significant inhibitory effect of LEAP2 on Ghrelin-induced food intake but no change in glucose levels. This suggests that the opposite effect between LEAP-2 and Ghrelin is not dependent on GH levels. The effect of LEAP2 on glucose levels was only observed in obese animals, which may be due to the fact that obese animals exhibit a state of hyperglycemia and insulin resistance, and therefore have a higher setting to trigger a counter-regulatory response to prevent hypoglycemia after LEAP-2 administration (108).On the one hand, GHSR increased expression in peptide cells of people and mice pancreatic islets (109). On the other hand, many studies have shown that both endogenous and exogenous Ghrelin can inhibit insulin production in mice, rats, and humans (110–113). Bayle et al. confronted isolated islets of Langerhans from rat pancreas to glucose with or without LEAP2 and ghrelin, and showed by measuring insulin production that Leap2 exerts modulation of insulin by blocking the insulin-inhibitory effect of Ghrelin (114). Similarly, M’Kadmi et al. demonstrated that N-terminal Leap2 _21-12_ blocked the inhibitory effect of Ghrelin on insulin secretion in rat pancreatic islet cells cultured *in vitro (* 95). Furthermore, overexpression of Leap2 in mice reduced blood glucose levels (94). Thus, circulating levels of Leap2 may influence glycemic control by blocking Ghrelin function to modulate insulin secretion. Recent studies have found that Leap2 _38-47_ exhibits insulin-promoting properties in cultured human pancreatic islet cells. The insulin-promoting properties are consistent with the LEAP2 fragment (Leap2 _38-47_) acting as a reverse Ghrelin receptor agonist (115). These impacts of Ghrelin are mediated at least in part by direct GHSR interactions that are differentially localized in α-cells, β-cells, δ-cells secreting growth inhibitory hormone (SST), and γ-cells of the pancreas expressing pancreatic polypeptide (pp) (109). *In vitro*, pharmacological and genetic inhibition of islet-derived ghrelin significantly enhances glucose-induced insulin response. In mice with modest obesity brought on by a high-fat diet, ghrelin deprivation increased insulin release and prevented decreased glucose tolerance (116). Leap2 thereby inhibits the insulin-suppressing and glucose-increasing actions of the ghrelin-GHSR pathway and might offer a therapeutic application for the control of T2DM.

## 5 Pcsk9

Pcsk9 (proprotein convertase subtilisin/kexin), has just come to light as one of the most important hepatokines, which induces the breakdown of hepatic low-density lipoprotein receptor (LDL-R) *via* the ribosomal/lysosomal pathway, thereby increasing circulating low-density lipoprotein cholesterol (LDL-C) levels (117, 118). It is expressed to a much lesser extent in the pancreas, adipose cells, gut, and kidney than it is in the liver, which has a high expression of it (119). According to the findings of a clinical investigation, a high-fructose diet elevated plasma Pcsk9 concentrations by 28% in healthy subjects and by 34% in the progeny of patients with T2DM who were more likely to be insulin resistant (120). As a result, decreasing plasma levels of PCSK9 presents itself as an intriguing possible treatment target for dyslipidemia in diabetic patients. Interestingly, feeding induced an increase in hepatic PCSK9 levels. Due to the increased insulin levels during feeding, it leads to the activation of Pcsk9 transcription by SREBP-1c (121). Given this characteristic, Pcsk9 is also referred to as feeding-induced hepatokine. Thus, we suggest that feeding-induced hepatokine Pcsk9 plays a role in T2DM. In this chapter, we describe in detail the impact of feeding-induced hepatokine Pcsk9 on the development of T2DM by acting on adipose tissue and the pancreas to improve glucolipid metabolism.

### 5.1 Effect of Pcsk9 on lipid metabolism

WAT malfunction and IR are thought to contribute significantly in the development of T2D, which delays clearance of triglyceride-rich lipoproteins (TRL), promotes elevated plasma TG and NEFA and flow to other peripheral tissues, leading to apoB overproduction, systemic lipotoxicity, inflammation, IR, and hyperinsulinemia (122–124). Upregulation of LDL-R uptake is associated with abnormal adipocyte metabolic function and risk of diabetes mellitus. Subjects who had normal cholesterol levels but had lower plasma levels of PCSK9 and higher levels of LDL-R and CD36 on the surface of their WAT also exhibited higher levels of WAT NLRP3 inflammasome activity and T2D-related hazard indicators (125). It’s possible that this is because LDL causes a reduction in adipocyte activity. Consistently, native LDL reduced WAT function and inhibited preadipocyte differentiation and function in mice (126). Others have reported that oxidized low-density lipoprotein (OxLDL) inhibits adipocyte differentiation (127). however, this effect is dependent on CD36 (a native scavenger receptor for VLDL and LDL, oxLDL, and NEFA) (128, 129). Of note, NLRP3/IL-1β inflammatory pathway stimulation promotes WAT malfunction and T2D and is controlled by LDL-R and CD36. It was revealed that oxLDL in CD36-internalized macrophages (130) and oxLDL and native LDL in endothelial cells (131) enhance the NLRP3 inflammasome, resulting in the release of the pro-inflammatory cytokine white IL-1β, which impedes insulin signaling in multiple cells, including adipocytes, β-cells, and hepatocytes (132, 133). Demers et al. declared that Pcsk9 stimulates the breakdown of CD36 in the acidic compartment behind the endoplasmic reticulum through a proteasome-sensitive mechanism that contributes to reducing the uptake of fatty acids and the deposition of triglyceride in tissues (134). Furthermore, Pcsk9 also limits visceral adipogenesis by degrading adipose tissue VLDL-R and LDL-R (135, 136). In some populations, elevated Plasma apolipoprotein B (apo B) plasma counts can predict the incidence of T2D 3-10 years prior to the onset of T2D independently of traditional risk factors (137, 138). ApoB plasma level indicates the quantity of small, dense LDL particles (137). Higher plasma apoB/pcsk9 levels are related with risk indicators for WAT malfunction and T2D, including postprandial hypertriglyceridemia, IR, hyperinsulinemia, and increased plasma interleukin 1 receptor antagonist (IL-1ra), according to multiple research (139, 140). Recently, we discovered that this ratio was indeed linked to high expression of LDL-R and CD36 on the WAT surface as well as WAT malfunction, inflammation, and IR (141). Therefore, Pcsk9 may be beneficial in improving WAT malfunction, inflammation, and IR, thereby reducing the hazard of T2DM.

### 5.2 Impact of Pcsk9 on glucose metabolism

In pancreatic β-cells, cholesterol is an integral part of the cell membrane and is involved in controlling the physical properties of the cell membrane, thus influencing the distribution and the functionality of membrane proteins, as well as the formation and fusion of vesicles (142). Whereas cholesterol accumulation is mainly *via* LDL-R, Cholesterol overload in β-cell s is a mechanism that limits or destroys glucose-stimulated insulin secretion (GSIS) (143), and it is related to any genetic or pharmacological treatment that raises LDL-R expression. It is believed that factors that influence the homeostasis of cellular cholesterol metabolism can have an effect on the beta-cell activity as well as the development of diabetes (144). In contrast, Roehrich et al. showed that human lipoproteins play an important role in modulating the survivability of β-cells. Purified human VLDL and LDL induced increased apoptosis and decreased insulin transcript levels. Conversely, HDL effectively counteracts cell death through mechanisms such as stimulation of Akt/protein kinase B (Akt/protein kinase B) and inhibition of caspase-3 cleavage (145). These findings point to the possibility that changes in lipoproteins are linked to the beta-cell malfunction that is seen throughout the advancement of T2DM. Similarly, Cnop et al. found a series of lipid abnormalities in individuals having T2DM that associated with the accumulation of cholesterol and fatty acids in pancreatic β-cell s and may lead to islet degeneration (146). It has been shown that Pcsk9 reduces LDL-R, which in turn reduces cholesterol accumulation in pancreatic β-cells and promotes increased glucose-dependent insulin secretion (GSIS) (147). Furthermore, Mbikay et al. reported that male mice with Pcsk9 deletion above four months old had more LDL-R while having lesser insulin in their pancreas and showed hypoinsulinemia, hyperglycemia, and glucose intolerance (148). Thus, Pcsk9 prevents islet degeneration and promotes insulin secretion by limiting pancreatic β-cell cholesterol accumulation. Notably, circulating/liver-derived (the primary target of monoclonal antibodies) does not affect the β-cell function and insulin secretion (147). Ramin et al. found that neither exogenous PCSK9, Alirocumab, nor PCSK9 silencing significantly affected glucose-stimulated insulin secretion (GSIS) from pancreatic β-cells (149). Similarly, Peyot et al. demonstrated that Pcsk9 deficiency did not have any toxic effects on β-cell activity and glucose homeostasis in either the whole-body KO or βKO mouse models (150). According to these findings, anti-PCSK9 medications, which primarily target circulating Pcsk9, have only a minimal influence on the malfunction of β-cell s and the prevalence of diabetes. In conclusion, Pcsk9 improves T2DM by limiting pancreatic β-cell cholesterol overload, maintaining glucose metabolic homeostasis, and preventing β-cell malfunction.

## 6 Clinical consideration of feeding-induced hepatokines in T2DM

Plasma levels of adropin are lower in individuals diagnosed with T2DM, particularly those who are obese or overweight (151). Recently, Adropin has also been increasingly studied in relation to diabetes-related complications. In addition to its function as a marker of malfunctional endothelium cells, adropin also has a preventative effect on the occurrence and advancement of cardiovascular diseases (26, 27, 152). Elevated plasma adropin concentrations in male individuals with T2DM patients and those showing obesity who were treated with liraglutide can partially explain the cardiovascular benefits and protective effects (153). Adropin, as a potential anti-inflammatory factor (154), emerges as a potential biomarker for predicting the development of MAFLD in patients with T2DM (155) and diabetic kidney disease (DKD) (156). In addition, the therapeutic potential of adropin for T2DM is demonstrated by its effects on the activity of various elements of the endocrine system, including the adrenal cortex. It has been shown that adropin inhibits steroidogenesis and secretion of adrenocorticotropic hormones (e.g., cortisol and aldosterone) in HAC15 cells by binding to the GPR19 receptor and activating the TGF-β-dependent pathway (157). Cortisol, a glucocorticoid that raises blood sugar and reduces insulin secretion (158), has been shown in a clinical study to increase insulin resistance in patients with T2D when the HPA axis loses its ability to lower cortisol levels during hyperglycemia (159). Similarly, aldosterone has been associated with glucose intolerance and insulin resistance, and drugs related to mineralocorticoid receptor (MRs) antagonists have been used to improve insulin resistance and endothelial dysfunction (160). Furthermore, the CNS effects of adropin inhibition of drinking water were also associated with the expression of GPR19 receptors (161). Perhaps adropin plays an important act in the control of water content in the body by modulating the CNS, with a pivotal role in preventing the intake of additional fluids. This could have a positive effect in relieving renal load in patients with diabetic nephropathy. The current study did not identify the adropin receptor(s), and clarifying the receptor(s) for adropin has potential implications for the treatment of type 2 diabetes. Some researchers have suggested that the biological effects of adropin are obtained by direct binding to the G protein-coupled receptor GPR19 (22, 161, 162), but the study by Foster et al. failed to confirm that adropin interacts with GPR19 (163). However, it has been shown that Adropin is a meningeal-binding protein that interacts with NB-3. Adropin may be important for NB-3 recruitment, concentration, and Notch1 receptor binding, which in turn contributes to cerebellar development (164). In addition, adropin may exert its physiological effects by acting directly on neurons in the PVN (165). PVN is a key autonomic control center that plays an important role in the regulation of fluid balance (166), energy homeostasis (167), and cardiovascular regulation (168). These findings further solidify that Adropin has an endocrine function as a hepatokine and provide the framework needed to link its peripheral effects to its role in the central nervous system.

Recent studies suggest that MANF performs a crucial part in food consumption as well as energy homeostasis (169) and its involvement in the modulation of metabolic disorders. Multiple clinical research declared that there is an association between T2DM and circulating MANF levels. Serum MANF levels were elevated in newly diagnosed prediabetic and T2DM patients than in non-diabetic controls (170), while circulating MANF levels were significantly diminished in T2DM patients (49). This is because early in patients with T2DM, IR in the liver, skeletal muscle, and adipose tissue causes endoplasmic reticulum stress in these tissues, inducing MANF expression. The compensatory increase in MANF may act as a protective mechanism against endoplasmic reticulum stress-induced cellular damage against disease progression, but as the disease progresses, accompanied by prolonged glucotoxicity and/or lipotoxicity, MANF expression decreases, thereby exacerbating the illness. Additionally, the negative correlation of MANF with FBG and HbA1c was confirmed by the results (49). Therefore, MANF may be a new therapeutic candidate to protect the organism from lipotoxicity and glucotoxicity-induced endoplasmic reticulum stress.

Through its interaction with the growth hormone secretagogue receptor (GHSR), the hormone ghrelin is able to control not only the amount of food that is consumed but also the level of glucose in the blood (171). A recently discovered endogenous ligand of the GHSR is known as Leap2 (172). The reduction in serum ghrelin levels and the elevation of Leap2 levels in individuals with type 2 diabetes may represent a physiological compensation as a response to a positive energy balance to maintain a normal energy balance. Lowering the Ghrelin/Leap2 ratio in individuals with T2DM may lower the overactivation of the GHSR in obese patients, which in turn may restore normal energy homeostasis (96). This viewpoint is reinforced by a paper that showed improvements in obesity and diabetes when levels of acyl ghrelin were reduced, levels of Leap2 were increased, or GHSR activity was blocked (173). A recent clinical study has shown that exogenous LEAP2 reduces postprandial glucose and suppresses appetite in healthy men, and these effects may be mediated through the GHSR (174). Thus the discovery of the endogenous inverse agonist LEAP2 may reveal potential therapeutic targets for gastric hunger-related diseases, including type 2 diabetes and obesity, as it interacts with gastric hunger and is expressed at elevated levels after RYGB surgery (115). Notably, many gastrin/gastrinase targeted drugs, such as anti-gastrin L-RNA inducers (anti-gastrin vaccines), GHSR antagonists, GHSR inverse agonists, GOAT inhibitors, cyclized deacetyl-gastrin analogs, none of which have entered late-stage clinical trials for the treatment of obesity or type 2 diabetes due to uncertainty about their safety and/or efficacy in humans (175–178). Therefore, further studies are needed to confirm the safety of LEAP-2 based compounds.

PCSK9, an endogenous suppressor of the LDLR pathway, works by guiding the breakdown of LDLR to the lysosome (179). PCSK9 is thought to be a factor suggesting increased cardiovascular risk in T2DM (180). Therefore, commercially available PCSK9 inhibitors can lower circulating LDL-C, thereby treating dyslipidemia in T2DM (181, 182). Recent studies have shown that Patients who have prodromal diabetes but not yet T2DM lack plasma PCSK9 levels that can forecast their likelihood of developing T2DM (183). Circulating levels of PCSK9 are linked to dyslipidemia in T2DM, which we suggest is due to its unique physiological functions related to lipid metabolism, but its beneficial effects on metabolic organs cannot be ignored, and anti-PCSK9 treatments that focus on circulating PCSK9 have a minimal effect on the organs that are being targeted. To date, up to nine PCSK9 inhibition strategies have been or are being developed to either block it’s binding to LDLR or prevent its maturation, secretion, or synthesis (184). These therapies include the use of anti-PCSK9 monoclonal antibodies (e.g., two FDA-approved drugs: alirocumab and evolocumab) (185), antisense oligonucleotides (ASO), small interfering RNAs (siRNAs), vaccines and small molecules (186). In patients with type 2 diabetes and hypercholesterolemia or mixed dyslipidemia treated with statins, PCSK9 inhibitors significantly reduced LDL-C, non-HDL-C and apoB levels. In addition, favorable changes were observed in postprandial levels of celiac disease, VLDL-C, and LDL-C (187, 188). Therefore, inhibition of PCSK9 is a promising new way to improve dyslipidemia in patients with T2DM to prevent cardiovascular disease. It should be noted, however, that completed clinical trials have not shown adverse effects of PCSK9 inhibitors on the risk of diabetes, but the safety of the inhibitors should be validated in long-term randomized trials (189).

## 7 Conclusion and future perspective

The liver is a vital organ in the body’s reaction to alterations in nutritional condition because it performs a crucial part in glucose and lipid metabolism. This review summarizes the crosstalk of some feeding-induced hepatokines Adropin, Manf, Leap2, and Pcsk9 in the liver and extrahepatic tissues such as brain, adipose, heart, and pancreatic tissues, and by targeting these feeding-induced hepatokines is expected to be a possible therapy for T2DM to help in control and treatment.

Many recent studies have demonstrated the high sensitivity of the liver to metabolic changes during fasting and refeeding, and here we discuss the important role played by other hepatokines regulated during the feeding-fasting-refeeding cycle concerning energy and glucolipid metabolism. For instance, refeeding signals during intermittent fasting (IF) induce the liver to produce a release of pregnancy band protein (PZP), and circulating PZP binds to GRP78 on the cell surface *via* the p38 MAPK-ATF2 signaling pathway, increasing UCP1 expression in BAT. PZP acts as a key hepatokine regulating IF, triggering energy homeostasis *via* the liver/BAT axis (190). Another feeding-induced hepatokine Tsukushi (TSK), is also involved in regulating energy metabolism through the liver/BAT axis. TSK ablation enhances thermogenic gene expression in BAT and suppresses obesity-associated inflammation in the liver and adipose tissue. Meanwhile, TSK acts as a metabolic signal from the liver, balancing the activation of hypothalamic melanocortin circuits during feeding (191). Angptl8 is a key regulator of the liver clock in response to food. angptl8 is regulated by nutritional and hormonal factors, and feeding induces an increase in its levels (192). It has been reported that ANGPTL8 not only induces the expression of brown adipocyte markers (193) but also promotes subcutaneous white adipose tissue (SAT) browning under acute and chronic hypothermic conditions (194). Fibroblast growth factor 21 (FGF21) is known to be a hepatokine induced by fasting and is being pursued as a therapeutic target for diabetes and obesity due to its rapid and effective action in improving insulin sensitivity (195). However, several studies have also demonstrated that FGF21 maintains a presence and functional role even during feeding. The expression of the FGF21 gene is paradoxically regulated by fasting and feeding signals. On the one hand, two fasting signals, including PPARa and glucagon-PKA, increase the expression of FGF21 gene. On the other hand, glucose and xylitol, which are feeding signals, also induced FGF21 expression through ChREBP activation (196). Overall, expression of the human FGF21 gene is paradoxically independently regulated by fasting and feeding signals. These regulatory mechanisms suggest that FGF21 increases in response to nutritional crises, including starvation and overfeeding. Therefore, FGF21 levels are likely to be useful markers for determining our nutritional status. Additionally, recent studies have identified a novel fasting-induced hepatokine orosomucoid (ORM) 2 as a key regulator of hepatic *de novo* lipogenesis (DNL) production. ORM2 plays an important role in inhibiting lipogenesis and maintaining hepatic and systemic lipid homeostasis. Therefore, ORM2 and its analogs may provide a potential pharmacological treatment for dyslipidemia (197). All in all, most of the regulatory responses to diet initially occur in the liver, and hepatokines play a key role in maintaining nutritional homeostasis by regulating the metabolism of other organs as signal messengers from the liver.

Given the interaction between these feeding-induced hepatokines and multiple organs, further *in vivo* experiments are needed to investigate their relationship with glucolipid metabolism, energy homeostasis, and inflammation, thus presenting novel approaches for the clinical management of diabetes in the years to come. Important areas of future research include (1) understanding how preclinical evidence of feeding-induced hepatokines translates to human studies, (2) determining the mechanisms by which feeding-induced hepatokines and other secreted factors integrate to modulate metabolism through interorgan interactions, (3) determining the pathology of these hepatokines in the development of diabetes physiology, will help to improve the prevention and even the treatment of this disease.

We hope that this will systematize the knowledge of feeding-induced hepatokines and help establish new lines of research regarding their role in metabolic organs.

## Author contributions

X-HX and Z-ZL contributed to the design of this article. Three authors R-BC, Q-YW and Y-YW conducted a literature search and research selection in Pubmed. R-BC and Q-YW wrote the manuscript and made final revisions. The work was done under the supervision of X-HX and Z-ZL. All authors contributed to the article and approved the submitted version.
