# Supplementary material for: Feeding-induced hepatokines and crosstalk with multi-organ: A novel therapeutic target for Type 2 diabetes
Source: Front Endocrinol (Lausanne). 2023 Mar 3;14:1094458. doi: 10.3389/fendo.2023.1094458 (PMC10020511; doi:10.3389/fendo.2023.1094458)
Supplement: Supplementary file 1 [file Table_1.doc]

**Table 1. F****eeding-induced hepatokines and their role in Type 2 diabetes**

| **Hepatokines** | **Target organs** | **Metabolic Roles** | **Human serum concentration in T2DM** | **Reference** |
| --- | --- | --- | --- | --- |
| **Adropin** | Liver, adipose tissue,pancreas,cardiac tissue | Harmonizes liver lipid metabolism and circadian rhythms；Increases cardiac glucose oxidation and insulin sensitization；Controls of adipose tissue formation；Regulates of pancreatic lipid metabolism and insulin secretion | Decreased | (11,20-34,35-41) |
| **Manf** | Liver,pancreas,  brain | Inhibits liver lipid deposition and inflammation；Protects and proliferates pancreatic beta cells; Regulation of energy metabolism through the CNS | Decreased | (45, 55-67，72-76,77-80，82-85) |
| **Leap2** | Liver,pancreas,  brain | Inhibits the liver, brain and pancreas actions of the ghrelin-GHSR system, including food intake, inhibition of insulin secretion and blood glucose elevation | Increased | （94, 95, 98，102-106，107，108，114，115） |
| **Pcsk9** | Liver, adipose tissue, pancreas | Degrades liver LDL-R and increases circulating LDL-C levels；Improves adipose tissue and pancreatic beta-cell dysfunction and regulates glucolipid metabolism | Increased | （125，134-136，139-141，147-150） |

Reference：

1. Kumar KG, Trevaskis JL, Lam DD, Sutton GM, Koza RA, Chouljenko VN, et al. Identification of adropin as a secreted factor linking dietary macronutrient intake with energy homeostasis and lipid metabolism. Cell Metab (2008) 8(6):468-481.10.1016/j.cmet.2008.10.011

20. Buchanan J, Mazumder PK, Hu P, Chakrabarti G, Roberts MW, Yun UJ, et al. Reduced cardiac efficiency and altered substrate metabolism precedes the onset of hyperglycemia and contractile dysfunction in two mouse models of insulin resistance and obesity. Endocrinology (2005) 146(12):5341-5349.10.1210/en.2005-0938

21. Altamimi TR, Gao S, Karwi QG, Fukushima A, Rawat S, Wagg CS, et al. Adropin regulates cardiac energy metabolism and improves cardiac function and efficiency. Metabolism (2019) 98:37-48.10.1016/j.metabol.2019.06.005

22. Thapa D, Stoner MW, Zhang M, Xie B, Manning JR, Guimaraes D, et al. Adropin regulates pyruvate dehydrogenase in cardiac cells via a novel GPCR-MAPK-PDK4 signaling pathway. Redox Biol (2018) 18:25-32.10.1016/j.redox.2018.06.003

23. Thapa D, Xie B, Zhang M, Stoner MW, Manning JR, Huckestein BR, et al. Adropin treatment restores cardiac glucose oxidation in pre-diabetic obese mice. J Mol Cell Cardiol (2019) 129:174-178.10.1016/j.yjmcc.2019.02.012

24. Gao S, McMillan RP, Jacas J, Zhu Q, Li X, Kumar GK, et al. Regulation of substrate oxidation preferences in muscle by the peptide hormone adropin. Diabetes (2014) 63(10):3242-3252.10.2337/db14-0388

25. Gao S, McMillan RP, Zhu Q, Lopaschuk GD, Hulver MW, Butler AA. Therapeutic effects of adropin on glucose tolerance and substrate utilization in diet-induced obese mice with insulin resistance. Mol Metab (2015) 4(4):310-324.10.1016/j.molmet.2015.01.005

26. Topuz M, Celik A, Aslantas T, Demir AK, Aydin S, Aydin S. Plasma adropin levels predict endothelial dysfunction like flow-mediated dilatation in patients with type 2 diabetes mellitus. J Investig Med (2013) 61(8):1161-1164.10.2310/JIM.0000000000000003

27. Lovren F, Pan Y, Quan A, Singh KK, Shukla PC, Gupta M, et al. Adropin is a novel regulator of endothelial function. Circulation (2010) 122(11 Suppl):S185-192.10.1161/CIRCULATIONAHA.109.931782

28. Kwon OS, Andtbacka RHI, Hyngstrom JR, Richardson RS. Vasodilatory function in human skeletal muscle feed arteries with advancing age: the role of adropin. J Physiol (2019) 597(7):1791-1804.10.1113/JP277410

29. Lian W, Gu X, Qin Y, Zheng X. Elevated plasma levels of adropin in heart failure patients. Intern Med (2011) 50(15):1523-1527.10.2169/internalmedicine.50.5163

30. Yosaee S, Soltani S, Sekhavati E, Jazayeri S. Adropin- A Novel Biomarker of Heart Disease: A Systematic Review Article. Iran J Public Health (2016) 45(12):1568-1576.

31. Mushala BAS, Scott I. Adropin: a hepatokine modulator of vascular function and cardiac fuel metabolism. Am J Physiol Heart Circ Physiol (2021) 320(1):H238-H244.10.1152/ajpheart.00449.2020

32. Sato K, Yamashita T, Shirai R, Shibata K, Okano T, Yamaguchi M, et al. Adropin Contributes to Anti-Atherosclerosis by Suppressing Monocyte-Endothelial Cell Adhesion and Smooth Muscle Cell Proliferation. Int J Mol Sci (2018) 19(5).10.3390/ijms19051293

33. Wu L, Fang J, Chen L, Zhao Z, Luo Y, Lin C, et al. Low serum adropin is associated with coronary atherosclerosis in type 2 diabetic and non-diabetic patients. Clin Chem Lab Med (2014) 52(5):751-758.10.1515/cclm-2013-0844

34. Zhao LP, You T, Chan SP, Chen JC, Xu WT. Adropin is associated with hyperhomocysteine and coronary atherosclerosis. Exp Ther Med (2016) 11(3):1065-1070.10.3892/etm.2015.2954

35. Celik A, Balin M, Kobat MA, Erdem K, Baydas A, Bulut M, et al. Deficiency of a new protein associated with cardiac syndrome X; called adropin. Cardiovasc Ther (2013) 31(3):174-178.10.1111/1755-5922.12025

36. Jasaszwili M, Wojciechowicz T, Billert M, Strowski MZ, Nowak KW, Skrzypski M. Effects of adropin on proliferation and differentiation of 3T3-L1 cells and rat primary preadipocytes. Mol Cell Endocrinol (2019) 496:110532.10.1016/j.mce.2019.110532

37. Jasaszwili M, Wojciechowicz T, Strowski MZ, Nowak KW, Skrzypski M. Adropin stimulates proliferation but suppresses differentiation in rat primary brown preadipocytes. Arch Biochem Biophys (2020) 692:108536.10.1016/j.abb.2020.108536

38. Jasaszwili M, Pruszynska-Oszmalek E, Wojciechowicz T, Strowski MZ, Nowak KW, Skrzypski M. Adropin Slightly Modulates Lipolysis, Lipogenesis and Expression of Adipokines but Not Glucose Uptake in Rodent Adipocytes. Genes (Basel) (2021) 12(6).10.3390/genes12060914

39. Zhang S, Chen Q, Lin X, Chen M, Liu Q. A Review of Adropin as the Medium of Dialogue between Energy Regulation and Immune Regulation. Oxid Med Cell Longev (2020) 2020:3947806.10.1155/2020/3947806

40. Chen S, Zeng K, Liu QC, Guo Z, Zhang S, Chen XR, et al. Adropin deficiency worsens HFD-induced metabolic defects. Cell Death Dis (2017) 8(8):e3008.10.1038/cddis.2017.362

41. Gyllenhammer LE, Lam J, Alderete TL, Allayee H, Akbari O, Katkhouda N, et al. Lower omental t-regulatory cell count is associated with higher fasting glucose and lower beta-cell function in adults with obesity. Obesity (Silver Spring) (2016) 24(6):1274-1282.10.1002/oby.21507

45. Tadimalla A, Belmont PJ, Thuerauf DJ, Glassy MS, Martindale JJ, Gude N, et al. Mesencephalic astrocyte-derived neurotrophic factor is an ischemia-inducible secreted endoplasmic reticulum stress response protein in the heart. Circ Res (2008) 103(11):1249-1258.10.1161/CIRCRESAHA.108.180679

55. Pakarinen E, Danilova T, Voikar V, Chmielarz P, Piepponen P, Airavaara M, et al. MANF Ablation Causes Prolonged Activation of the UPR without Neurodegeneration in the Mouse Midbrain Dopamine System. eNeuro (2020) 7(1).10.1523/ENEURO.0477-19.2019

56. Xu S, Di Z, He Y, Wang R, Ma Y, Sun R, et al. Mesencephalic astrocyte-derived neurotrophic factor (MANF) protects against Abeta toxicity via attenuating Abeta-induced endoplasmic reticulum stress. J Neuroinflammation (2019) 16(1):35.10.1186/s12974-019-1429-0

57. Wang D, Hou C, Cao Y, Cheng Q, Zhang L, Li H, et al. XBP1 activation enhances MANF expression via binding to endoplasmic reticulum stress response elements within MANF promoter region in hepatitis B. Int J Biochem Cell Biol (2018) 99:140-146.10.1016/j.biocel.2018.04.007

58. Wu T, Liu Q, Li Y, Li H, Chen L, Yang X, et al. Feeding-induced hepatokine, Manf, ameliorates diet-induced obesity by promoting adipose browning via p38 MAPK pathway. J Exp Med (2021) 218(6).10.1084/jem.20201203

59. Bourougaa K, Naski N, Boularan C, Mlynarczyk C, Candeias MM, Marullo S, et al. Endoplasmic reticulum stress induces G2 cell-cycle arrest via mRNA translation of the p53 isoform p53/47. Mol Cell (2010) 38(1):78-88.10.1016/j.molcel.2010.01.041

60. Brewer JW, Diehl JA. PERK mediates cell-cycle exit during the mammalian unfolded protein response. Proc Natl Acad Sci U S A (2000) 97(23):12625-12630.10.1073/pnas.220247197

61. Danilova T, Belevich I, Li H, Palm E, Jokitalo E, Otonkoski T, et al. MANF Is Required for the Postnatal Expansion and Maintenance of Pancreatic beta-Cell Mass in Mice. Diabetes (2019) 68(1):66-80.10.2337/db17-1149

62. Hakonen E, Chandra V, Fogarty CL, Yu NY, Ustinov J, Katayama S, et al. MANF protects human pancreatic beta cells against stress-induced cell death. Diabetologia (2018) 61(10):2202-2214.10.1007/s00125-018-4687-y

63. Lindahl M, Danilova T, Palm E, Lindholm P, Voikar V, Hakonen E, et al. MANF is indispensable for the proliferation and survival of pancreatic beta cells. Cell Rep (2014) 7(2):366-375.10.1016/j.celrep.2014.03.023

64. Cunha DA, Cito M, Grieco FA, Cosentino C, Danilova T, Ladriere L, et al. Pancreatic beta-cell protection from inflammatory stress by the endoplasmic reticulum proteins thrombospondin 1 and mesencephalic astrocyte-derived neutrotrophic factor (MANF). J Biol Chem (2017) 292(36):14977-14988.10.1074/jbc.M116.769877

65. Chen L, Feng L, Wang X, Du J, Chen Y, Yang W, et al. Mesencephalic astrocyte-derived neurotrophic factor is involved in inflammation by negatively regulating the NF-kappaB pathway. Sci Rep (2015) 5:8133.10.1038/srep08133

66. Yagi T, Asada R, Kanekura K, Eesmaa A, Lindahl M, Saarma M, et al. Neuroplastin Modulates Anti-inflammatory Effects of MANF. iScience (2020) 23(12):101810.10.1016/j.isci.2020.101810

67. Wu H, Li H, Wen W, Wang Y, Xu H, Xu M, et al. MANF protects pancreatic acinar cells against alcohol-induced endoplasmic reticulum stress and cellular injury. J Hepatobiliary Pancreat Sci (2021) 28(10):883-892.10.1002/jhbp.928

72. Sousa-Victor P, Neves J, Cedron-Craft W, Ventura PB, Liao CY, Riley RR, et al. MANF regulates metabolic and immune homeostasis in ageing and protects against liver damage. Nat Metab (2019) 1(2):276-290.10.1038/s42255-018-0023-6

73. He M, Wang C, Long XH, Peng JJ, Liu DF, Yang GY, et al. Mesencephalic astrocyte-derived neurotrophic factor ameliorates steatosis in HepG2 cells by regulating hepatic lipid metabolism. World J Gastroenterol (2020) 26(10):1029-1041.10.3748/wjg.v26.i10.1029

74. Zhang G, Liu Q, Li Y, Huang C, Zhou J, Zhao Y, et al. Mesencephalic astrocyte-derived neurotrophic factor alleviates alcohol induced hepatic steatosis via activating Stat3-mediated autophagy. Biochem Biophys Res Commun (2021) 550:197-203.10.1016/j.bbrc.2021.02.123

75. Zhang X, Heckmann BL, Campbell LE, Liu J. G0S2: A small giant controller of lipolysis and adipose-liver fatty acid flux. Biochim Biophys Acta Mol Cell Biol Lipids (2017) 1862(10 Pt B):1146-1154.10.1016/j.bbalip.2017.06.007

76. Liu J, Wu Z, Han D, Wei C, Liang Y, Jiang T, et al. Mesencephalic Astrocyte-Derived Neurotrophic Factor Inhibits Liver Cancer Through Small Ubiquitin-Related Modifier (SUMO)ylation-Related Suppression of NF-kappaB/Snail Signaling Pathway and Epithelial-Mesenchymal Transition. Hepatology (2020) 71(4):1262-1278.10.1002/hep.30917

77. Yang S, Yang H, Chang R, Yin P, Yang Y, Yang W, et al. MANF regulates hypothalamic control of food intake and body weight. Nat Commun (2017) 8(1):579.10.1038/s41467-017-00750-x

78. Wang H, Ke Z, Alimov A, Xu M, Frank JA, Fang S, et al. Spatiotemporal expression of MANF in the developing rat brain. PLoS One (2014) 9(2):e90433.10.1371/journal.pone.0090433

79. Cakir I, Nillni EA. Endoplasmic Reticulum Stress, the Hypothalamus, and Energy Balance. Trends Endocrinol Metab (2019) 30(3):163-176.10.1016/j.tem.2019.01.002

80. Tang Q, Liu Q, Li J, Yan J, Jing X, Zhang J, et al. MANF in POMC Neurons Promotes Brown Adipose Tissue Thermogenesis and Protects Against Diet-Induced Obesity. Diabetes (2022) 71(11):2344-2359.10.2337/db21-1128

82. Danilova T, Galli E, Pakarinen E, Palm E, Lindholm P, Saarma M, et al. Mesencephalic Astrocyte-Derived Neurotrophic Factor (MANF) Is Highly Expressed in Mouse Tissues With Metabolic Function. Front Endocrinol (Lausanne) (2019) 10:765.10.3389/fendo.2019.00765

83. Bartke A, Sun LY, Longo V. Somatotropic signaling: trade-offs between growth, reproductive development, and longevity. Physiol Rev (2013) 93(2):571-598.10.1152/physrev.00006.2012

84. Furigo IC, Teixeira PDS, de Souza GO, Couto GCL, Romero GG, Perello M, et al. Growth hormone regulates neuroendocrine responses to weight loss via AgRP neurons. Nat Commun (2019) 10(1):662.10.1038/s41467-019-08607-1

85. Zhong C, Song Y, Wang Y, Zhang T, Duan M, Li Y, et al. Increased food intake in growth hormone-transgenic common carp (Cyprinus carpio L.) may be mediated by upregulating Agouti-related protein (AgRP). Gen Comp Endocrinol (2013) 192:81-88.10.1016/j.ygcen.2013.03.024

94. Ge X, Yang H, Bednarek MA, Galon-Tilleman H, Chen P, Chen M, et al. LEAP2 Is an Endogenous Antagonist of the Ghrelin Receptor. Cell Metab (2018) 27(2):461-469 e466.10.1016/j.cmet.2017.10.016

95. M'Kadmi C, Cabral A, Barrile F, Giribaldi J, Cantel S, Damian M, et al. N-Terminal Liver-Expressed Antimicrobial Peptide 2 (LEAP2) Region Exhibits Inverse Agonist Activity toward the Ghrelin Receptor. J Med Chem (2019) 62(2):965-973.10.1021/acs.jmedchem.8b01644

98. Islam MN, Mita Y, Maruyama K, Tanida R, Zhang W, Sakoda H, et al. Liver-expressed antimicrobial peptide 2 antagonizes the effect of ghrelin in rodents. J Endocrinol (2020) 244(1):13-23.10.1530/JOE-19-0102

102. Lopez Soto EJ, Agosti F, Cabral A, Mustafa ER, Damonte VM, Gandini MA, et al. Constitutive and ghrelin-dependent GHSR1a activation impairs CaV2.1 and CaV2.2 currents in hypothalamic neurons. J Gen Physiol (2015) 146(3):205-219.10.1085/jgp.201511383

103.Mustafa ER, Cordisco Gonzalez S, Damian M, Cantel S, Denoyelle S, Wagner R, et al. LEAP2 Impairs the Capability of the Growth Hormone Secretagogue Receptor to Regulate the Dopamine 2 Receptor Signaling. Front Pharmacol (2021) 12:712437.10.3389/fphar.2021.712437

104.Cornejo MP, Castrogiovanni D, Schioth HB, Reynaldo M, Marie J, Fehrentz JA, et al. Growth hormone secretagogue receptor signalling affects high-fat intake independently of plasma levels of ghrelin and LEAP2, in a 4-day binge eating model. J Neuroendocrinol (2019) 31(10):e12785.10.1111/jne.12785

105.Shankar K, Metzger NP, Singh O, Mani BK, Osborne-Lawrence S, Varshney S, et al. LEAP2 deletion in mice enhances ghrelin's actions as an orexigen and growth hormone secretagogue. Mol Metab (2021) 53:101327.10.1016/j.molmet.2021.101327

106.Shen M, Manca C, Suriano F, Nallabelli N, Pechereau F, Allam-Ndoul B, et al. Three of a Kind: Control of the Expression of Liver-Expressed Antimicrobial Peptide 2 (LEAP2) by the Endocannabinoidome and the Gut Microbiome. Molecules (2021) 27(1).10.3390/molecules27010001

107.Al-Massadi O, Muller T, Tschop M, Dieguez C, Nogueiras R. Ghrelin and LEAP-2: Rivals in Energy Metabolism. Trends Pharmacol Sci (2018) 39(8):685-694.10.1016/j.tips.2018.06.004

108.Lugilde J, Casado S, Beiroa D, Cunarro J, Garcia-Lavandeira M, Alvarez CV, et al. LEAP-2 Counteracts Ghrelin-Induced Food Intake in a Nutrient, Growth Hormone and Age Independent Manner. Cells (2022) 11(3).10.3390/cells11030324

114. Bayle M, Peraldi-Roux S, Gautheron G, Cros G, Oiry C, Neasta J. Liver-Expressed Antimicrobial Peptide 2 antagonizes the insulinostatic effect of ghrelin in rat isolated pancreatic islets. Fundam Clin Pharmacol (2022) 36(2):375-377.10.1111/fcp.12722

115. Hagemann CA, Zhang C, Hansen HH, Jorsal T, Rigbolt KTG, Madsen MR, et al. Identification and Metabolic Profiling of a Novel Human Gut-derived LEAP2 Fragment. J Clin Endocrinol Metab (2021) 106(2):e966-e981.10.1210/clinem/dgaa803

125.Cyr Y, Lamantia V, Bissonnette S, Burnette M, Besse-Patin A, Demers A, et al. Lower plasma PCSK9 in normocholesterolemic subjects is associated with upregulated adipose tissue surface-expression of LDLR and CD36 and NLRP3 inflammasome. Physiol Rep (2021) 9(3):e14721.10.14814/phy2.14721

134.Demers A, Samami S, Lauzier B, Des Rosiers C, Ngo Sock ET, Ong H, et al. PCSK9 Induces CD36 Degradation and Affects Long-Chain Fatty Acid Uptake and Triglyceride Metabolism in Adipocytes and in Mouse Liver. Arterioscler Thromb Vasc Biol (2015) 35(12):2517-2525.10.1161/ATVBAHA.115.306032

135.Roubtsova A, Munkonda MN, Awan Z, Marcinkiewicz J, Chamberland A, Lazure C, et al. Circulating proprotein convertase subtilisin/kexin 9 (PCSK9) regulates VLDLR protein and triglyceride accumulation in visceral adipose tissue. Arterioscler Thromb Vasc Biol (2011) 31(4):785-791.10.1161/ATVBAHA.110.220988

136.Baragetti A, Balzarotti G, Grigore L, Pellegatta F, Guerrini U, Pisano G, et al. PCSK9 deficiency results in increased ectopic fat accumulation in experimental models and in humans. Eur J Prev Cardiol (2017) 24(17):1870-1877.10.1177/2047487317724342

139.Wassef H, Bissonnette S, Saint-Pierre N, Lamantia V, Cyr Y, Chretien M, et al. The apoB-to-PCSK9 ratio: A new index for metabolic risk in humans. J Clin Lipidol (2015) 9(5):664-675.10.1016/j.jacl.2015.06.012

140.Bissonnette S, Saint-Pierre N, Lamantia V, Cyr Y, Wassef H, Faraj M. Plasma IL-1Ra: linking hyperapoB to risk factors for type 2 diabetes independent of obesity in humans. Nutr Diabetes (2015) 5:e180.10.1038/nutd.2015.30

141.Cyr Y, Bissonnette S, Lamantia V, Wassef H, Loizon E, Ngo Sock ET, et al. White Adipose Tissue Surface Expression of LDLR and CD36 is Associated with Risk Factors for Type 2 Diabetes in Adults with Obesity. Obesity (Silver Spring) (2020) 28(12):2357-2367.10.1002/oby.22985

147.Da Dalt L, Ruscica M, Bonacina F, Balzarotti G, Dhyani A, Di Cairano E, et al. PCSK9 deficiency reduces insulin secretion and promotes glucose intolerance: the role of the low-density lipoprotein receptor. Eur Heart J (2019) 40(4):357-368.10.1093/eurheartj/ehy357

148.Mbikay M, Sirois F, Mayne J, Wang GS, Chen A, Dewpura T, et al. PCSK9-deficient mice exhibit impaired glucose tolerance and pancreatic islet abnormalities. FEBS Lett (2010) 584(4):701-706.10.1016/j.febslet.2009.12.018

149.Ramin-Mangata S, Thedrez A, Nativel B, Diotel N, Blanchard V, Wargny M, et al. Effects of proprotein convertase subtilisin kexin type 9 modulation in human pancreatic beta cells function. Atherosclerosis (2021) 326:47-55.10.1016/j.atherosclerosis.2021.03.044

150.Peyot ML, Roubtsova A, Lussier R, Chamberland A, Essalmani R, Murthy Madiraju SR, et al. Substantial PCSK9 inactivation in beta-cells does not modify glucose homeostasis or insulin secretion in mice. Biochim Biophys Acta Mol Cell Biol Lipids (2021) 1866(8):158968.10.1016/j.bbalip.2021.158968
